# Predictors of Transition from Mild Cognitive Impairment to Normal Cognition and Dementia

**DOI:** 10.3390/bs15111552

**Published:** 2025-11-14

**Authors:** Jiage Gao, Lin Liu, Zifeng Yang, Jialing Fan, for the Alzheimer’s Disease Neuroimaging Initiative

**Affiliations:** 1 Institute of Psychological and Brain Sciences, Liaoning Normal University, Dalian 116029, China; 2 Laboratory of Brain and Cognitive Neuroscience, Liaoning Province, Dalian 116029, China

**Keywords:** mild cognitive impairment, transition, prediction, cognitive measure, hippocampus, multinomial logistic regression

## Abstract

Mild cognitive impairment (MCI) represents a heterogeneous state between normal aging and dementia, with varied transition pathways. While factors influencing MCI progression are known, their role in cognitive reversal is unclear. This study analyzed 756 Alzheimer’s Disease Neuroimaging Initiative (ADNI) participants, classified as progressive MCI (pMCI, N = 272, mean age = 75.10 ± 7.34 years), reversible MCI (rMCI, N = 52, mean age = 69.94 ± 7.98 years) and stable MCI (sMCI, N = 432, mean age = 73.34 ± 7.44 years) based on 36-month follow-up. We compared demographic, lifestyle, clinical, cognitive, neuroimaging, and biomarker data across groups and developed a prediction model. Patients in the rMCI group were significantly younger and had a higher level of education compared with those in the pMCI group. Memory, general cognition, daily functional activities, and hippocampal volume effectively distinguished all three groups. In contrast, Aβ, tau, and other brain regions were able to distinguish only between progressive and non-progressive cases. Informant-reported Everyday Cognition (Ecog) scales outperformed self-reported Ecog scales in differentiating subtypes and predicting progression. Multinomial regression revealed that higher education, larger hippocampal volume, and lower daily functional impairment were associated with reversion, whereas *APOE ε4*, poorer memory, and greater brain atrophy predicted progression (model accuracy: 78%). The results confirm the significant utility of hippocampal volume, education level, and daily functional activities for assessing baseline disparities and predicting reversion. This study highlights the differential contributions of cognitive abilities and brain regions on MCI reversal, advancing understanding of MCI heterogeneity and providing evidence for precise diagnosis and treatment in early MCI.

## 1. Introduction

Dementia is a prevalent neurodegenerative disorder among the elderly population. While several interventions and treatment approaches currently exist—including symptomatic therapies and emerging disease-modifying agents—they primarily slow disease progression rather than providing a cure. There remains a long path ahead before we can truly realize the goal of curing dementia (Fox et al., 2025; Zhi et al., 2025). The transitional state between normal cognitive aging and dementia is defined as mild cognitive impairment (MCI) (Albert et al., 2011; Luo et al., 2022; Petersen, 2004). However, MCI exhibits marked heterogeneity. While MCI represents a high-risk state for dementia development, not all individuals will progress (Lopez et al., 2012). Based on the future developmental trends of MCI, patients may progress to dementia (progressive MCI, pMCI), revert to normal cognition (NC) (reversible MCI, rMCI), or remain in a stable condition (stable MCI, sMCI) (Iraniparast et al., 2022; Petersen et al., 2001). According to the recent studies, the transition rate from MCI to NC has been reported to range from 4.5% to 30%, which may reflect differences in clinical versus community-based screening methods as well as variations in follow-up duration (Koepsell & Monsell, 2012; Malek-Ahmadi, 2016; Nordlund et al., 2010; Sha et al., 2022; Wood, 2016; Xue et al., 2019; Yu et al., 2025). These findings suggest that a substantial proportion of MCI patients have the potential to revert to NC. Therefore, targeting therapeutic interventions on rMCI may represents a promising strategy to optimize MCI management and to delay or even prevent the onset of dementia.

The progression of MCI is influenced by a multitude of factors. For example, older age, a higher number of risk alleles, lower educational levels, poorer baseline cognitive function and psychological factors (e.g., depression) are all associated with a greater likelihood of MCI progression (Li et al., 2016; Marquié et al., 2023; Nakahata et al., 2021; Pink et al., 2015; Serrano-Pozo et al., 2021; Tucker-Drob, 2019). Nevertheless, the roles of these factors in MCI reversal remain unclear. Previous studies have demonstrated significant differences in these factors between progressive and non-progressive groups (W. Wang et al., 2023). Few studies have investigated whether stable and reversible subtypes can be distinguished within the non-progressive group. Specifically, previous studies have reported that age—considered one of the most critical predictors of MCI progression—carries the greatest weight in progression-related risk models. However, its predictive utility in cognitive reversal remains uncertain (Hu et al., 2017). While some evidence suggests that younger individuals have a higher likelihood of reverting to normal cognitive status (Pandya et al., 2017), other studies indicate that the predictive influence of age on progression cannot be directly extrapolated to predict reversal (M. Wang et al., 2023). APOE (apolipoprotein E) is a polymorphic protein, and the *APOE ε4 allele* is a well-established genetic risk factor for late-onset Alzheimer’s disease (AD), significantly increases the likelihood of developing AD (Serrano-Pozo et al., 2021). Thus, carriers of the *APOE ε4* allele exhibit a markedly higher risk of progression to AD compared to non-carriers (Scarabino et al., 2016). However, whether ε4 non-carriers are more likely to experience cognitive reversal remains an open question. Some studies have reported that fewer *APOE ε4 alleles* are associated with a higher probability of cognitive reversal (Caselli et al., 2009), whereas others suggest that having fewer *APOE ε4 alleles* may reduce progression risk without directly predicting reversal (Ward et al., 2020). Educational attainment is closely associated with cognitive reserve, which, in turn, may influences the progression trajectory of MCI (Iraniparast et al., 2022; Tucker-Drob, 2019). Higher cognitive reserve may delay the progression from MCI to AD, but its role in cognitive reversal is not yet well established (Mazzeo et al., 2019; H. Xu et al., 2020). Furthermore, baseline cognitive status is a well-established predictor: lower baseline scores strongly predict progression to dementia, while higher baseline cognitive performance increases the likelihood of reversal (Grassi et al., 2019; Jack et al., 2010). Neuroimaging markers (e.g., gray matter atrophy rates) have been extensively validated as bidirectional markers of cognitive change and demonstrate significant heterogeneity across distinct MCI subtype, showing moderate predictive utility for both disease progression and reversion (Lissek & Suchan, 2021; Luo et al., 2022; Pyun et al., 2021; Serrano-Pozo et al., 2021). Nevertheless, their predictive accuracy requires further elucidation. For instance, although hippocampal atrophy is a well-established neuroimaging biomarker of AD, it remains unclear whether preserved hippocampal volume can indicate better cognitive performance in individuals exhibiting cognitive reversal (Yang et al., 2022).

Additionally, non-neurodegenerative conditions can also influence MCI conversion. In terms of lifestyle factors, individuals with a history of smoking, alcohol use are at significantly higher risk of MCI progression (Durazzo et al., 2018; W. Xu et al., 2017). Similarly, dietary habits and activities are also influential factors that should not be overlooked. Consumption of fresh fruits is associated with an increased rate of reversion from MCI, while cognitively stimulating activities (e.g., reading) not only helps slow the decline of cognitive decline but also promote MCI reversion from MCI (Katayama et al., 2021; Sha et al., 2022). Marital status in later life reduces the risk of MCI and dementia, though no direct evidence currently links it to MCI reversion (Skirbekk et al., 2023). In terms of physiological and psychological factors, research has identified sleep disorders are a well-established risk factor for both MCI and dementia, and treating sleep disorders may lead to measurable improvements in cognitive function (Mayer et al., 2024). Depression, apathy, diabetes mellitus and hyperlipidemias also should not be overlooked when investigating the processes underlying MCI conversion mechanisms (Li et al., 2016). However, the absence of such lifestyle factors or medical conditions may only serve to slow MCI progression rather than actively promote reversion (Sha et al., 2022). In summary, while these non-neurodegenerative factors are strongly associated with MCI progression, the majority of their correlation with cognitive reversion remains unclear. Moreover, the presence of multiple risk factors—which may interact with each other—makes it difficult to determine the individual predictive power of each factor.

Therefore, in this study, we categorized MCI participants into three groups—pMCI, rMCI, and sMCI—based on data from the Alzheimer’s Disease Neuroimaging Initiative (ADNI) database. We aimed to systematically investigate the characteristic differences among these subpopulations within a unified analytical framework, delineate the distinct features of each MCI subgroup, and develop a prediction model of whether baseline MCI status is likely to transition.

## 2. Methods

### 2.1. Participant Characteristics

Participants in this study were drawn from ADNI (http://adni.loni.usc.edu/). ADNI is a longitudinal multicenter study that has recruited participants aged 55+ years, including individuals with NC, MCI, and AD. It collects comprehensive multimodal data covering neuroimaging, biomarkers, clinical assessments, and genetic profiles. Prior to data collection, ADNI obtained approval from the Institutional Review Board (IRB) of each participating site and written informed consent from all its participants or their legally authorized representatives. 

All participants included in the present study had a baseline diagnosis of MCI. In the ADNI database, the diagnostic criteria for MCI include: (1) Subjective Memory Concern; (2) Objective Cognitive Impairment, determined via standardized cognitive assessments, with the following specifications: (i) a Clinical Dementia Rating (CDR) (Berg, 1988) score of 0.5 and Mini-Mental State Examination (MMSE) (Folstein et al., 1975), scores between 24–30; (ii) intact activities of daily living; and (iii) objective memory loss measured by education adjusted scores.

Participants fulfilling both criteria were diagnosed with MCI.

MCI participants were further subclassified based on their cognitive trajectories over a 36-month follow-up period, using the following criteria:

Progressive MCI (pMCI): Individuals who experienced cognitive decline during the follow-up, meeting the threshold for a diagnosis of dementia. These participants transitioned from MCI to a clinical diagnosis of AD or other kinds of dementia.

Reversible MCI (rMCI): Individuals whose cognitive function improved during the follow-up, returning to a normal cognitive range and no longer meeting the criteria for MCI.

Stable MCI (sMCI): Individuals who maintained relatively stable cognitive performance throughout the follow-up period, with no significant improvement or deterioration.

After initially selecting MCI participants who exhibited progression, reversion, or remained stable over 36-month follow-up, we specifically verified the completeness of their demographic data and key genetic variable, namely age, sex, education level, and number of *APOE ε4 alleles*. Only participants with complete data for all four variables were included in the final analysis. Finally, total of 756 participants from ADNI-1, ADNI-GO, ADNI-2, and ADNI-3 were included in this study.

### 2.2. Demographic Variables

Demographic variables included age, sex, years of education, and the number of *APOE ε4 alleles* (0/1/2). Marital status was categorized as either living with a partner (married) or living alone (not married/divorced/widowed).

### 2.3. Lifestyle and Clinical Characteristics

Lifestyle variables included living arrangement, obesity status, smoking history, alcohol using history. Body Mass Index (BMI) was calculated using height and weight; individuals with a BMI ≥ 28 were classified as obese, while those with lower BMI were considered non-obese. Clinical characteristics consisted of medical history variables (Neurological & Psychiatric Disorders/Cardiovascular Diseases/Hypertension/Stroke/Respiratory Diseases/Endocrine Disorders/Major Surgical History), and Geriatric Depression Scale (GDS) (Sheikh, 1986) was used for assessing depressive symptoms.

### 2.4. Behavioral Data

Cognitive assessments covered five domains. General Cognition: CDR Sum of Boxes (CDRSB), MMSE, Alzheimer’s Disease Assessment Scale-Cognitive Subscale (ADAS-cog) (Rosen et al., 1984); Memory: Rey Auditory Verbal Learning Test (RAVLT) (Rey, 1958), Logical Memory Delayed Recall Total (LDELtotal) (Wechsler, 1987); Executive Function: Trail Making Test (TMT) (Reitan, 1958) Daily Function: Functional Activities Questionnaire (FAQ) (Pfeffer et al., 1982); and Language: Category Fluency (Animal Naming) Test (CFT) (Butters et al., 1987). For the TMT, Part B–Part A difference scores were calculated. The Everyday Cognition (Ecog) (Farias et al., 2008) was administered to assess patient and caregiver ratings across distinct cognitive domains, capturing self-reported (participant) and informant-reported (study partner) perspectives on functional abilities. In general, study partners are someone who knows the patient well, such as caregivers.

### 2.5. Neuroimaging Data and Biomarkers

Neuroimaging data from the ADNI database included volumetric measurements of the whole brain and five specific brain regions: ventricles, hippocampus, entorhinal cortex, fusiform gyrus, and middle temporal gyrus. Biomarkers data included cerebrospinal fluid (CSF) tau, p-tau and Aβ datas.

### 2.6. Statistical Analyses

In the analysis of demographic characteristics, categorical variables such as sex and *APOE ε4 allele* count were analyzed using the chi-square test, while continuous variables such as age and education were analyzed using One-Way Analysis of Variance (one-way ANOVA). Additionally, non-parametric tests were conducted to examine the distribution of age and education.

A general linear model (GLM) was applied to all behavioral data, brain region volumes, and biomarker measurements treated as continuous variables to assess group differences. Age, sex, years of education, and the number of *APOE ε4 alleles* were included as covariates. For brain region data, total intracranial volume (TIV) was additionally included as a covariate. Except for BMI and GDS, all variables related to medical history, lifestyle, and clinical characteristics were binary and analyzed using the chi-square test. BMI and GDS were analyzed using one-way ANOVA. Additionally, a mixed-design ANOVA was conducted on the Ecog data, with source (self vs. informant) as the within-subjects factor, group (pMCI, rMCI, sMCI) as the between-subjects factor, and the Ecog subscales as the dependent variables. We evaluated self-reported and informant-reported subscores within the Ecog scale to determine whether these two types of reports significantly differed, and to assess their utility in predicting MCI reversion. All post hoc test was corrected using the Bonferroni method.

To investigate the relationship between neuroimaging data and behavioral data, partial correlation analyses were conducted in each group between the volumes of specific brain regions (ventricles, hippocampus, entorhinal cortex, fusiform gyrus, middle temporal gyrus) and cognitive measure, with age, sex, years of education, number of *APOE ε4 alleles*, and TIV included as covariates. The results were corrected for multiple comparisons using the false discovery rate (FDR) method.

Predictors of MCI transformation were examined using multinomial logistic regression. The model was adjusted for age, sex, education, and *APOE ε4* status. Significant predictors (*p* < 0.05 in univariable analyses) were then entered into the multivariable model, with final variable selection determined by likelihood ratio tests. In the regression model, the sMCI group was set as the reference category. All continuous variables underwent z-score, and regional brain volumes were normalized as percentages of total intracranial volume (TIV) prior to z-score transformation.

All analysis processes were conducted using SPSS 25.

## 3. Results

### 3.1. Demographic Characteristics

A total of 756 participants were included in the study (male: 449; female: 307), comprising 52 were rMCI (male: 28; female: 24), 432 were sMCI (male: 265; female: 167), and 272 were pMCI (male: 156; female: 116). Significant differences were observed in the mean and distribution of age (*p*s < 0.05) and years of education (*p*s < 0.05) across groups (Table 1). Participants in the rMCI (*p* = 0.03) and sMCI (*p* = 0.033) groups were significantly younger than the pMCI group. In addition, the rMCI group had significantly higher education levels compared to pMCI (*p* = 0.007) and sMCI (*p* = 0.034).

Significant differences were observed in obesity levels (*p* = 0.001), with the sMCI group showing significantly higher values compared to the pMCI group. No significant differences were found in the total score of the GDS. Among the clinical characteristics, Only the presence of neurological conditions (*p* = 0.019) and stroke (*p* = 0.035) showed a significant difference.

Results from the GLM for behavioral data indicated significant between-group differences across all cognitive domains (*p*s < 0.05). Further analysis revealed that MMSE, RAVLT-immediate recall, RAVLT-delayed recall, and CFT followed a descending pattern across groups (rMCI > sMCI > pMCI). FAQ scores also significantly differentiated the three groups (rMCI < sMCI < pMCI). For CDRSB, LDELtotal and the TMT(B-A) difference, the pMCI group performed significantly worse than the rMCI and sMCI groups (Table 1).

### 3.2. Ecog Cognitive Test

**Figure 1:**
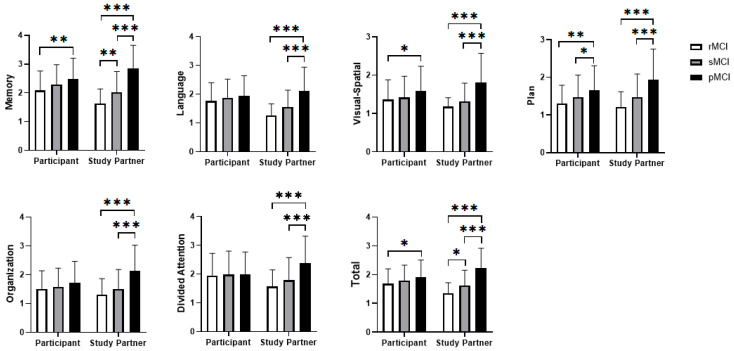
Differences between self-reported and informant-reported scales. Significance levels: * *p* < 0.05, ** *p* < 0.01, *** *p* < 0.001.

A mixed-design ANOVA was conducted to compare ratings between two sources (participants and study partners, as the within-subjects factor) and across three MCI groups (pMCI, rMCI, sMCI, as the between-subjects factor) on the Ecog scales. Significant main effects were observed across all subscales (Table 2, Figure 1). Group-wise analyses indicated that Memory and Total scale scores could differentiate all three groups, whereas other scale scores only distinguished the pMCI group the rMCI and sMCI groups. No significant main effect was observed for source (self vs. informant). However, a significant interaction between group and Ecog source suggested that group influenced the discrepancies between self and informant-assessments. Self-reported Language, Executive, and Attention did not differ significantly across groups, while other self-reported subscales showed at least one significant pairwise difference. In contrast, all informant-reported subscales significantly distinguished rMCI and sMCI from pMCI, with Memory and Total scores further differentiating rMCI from sMCI. These results indicate that informant ratings in these domains effectively distinguish among the three MCI subtypes (*p* < 0.05, Bonferroni-corrected).

### 3.3. Neuroanatomical Characteristics

**Figure 2:**
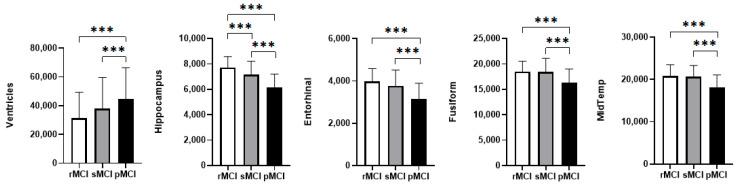
Group differences in brain region volumes among the three MCI subtypes. Significance levels: *** *p* < 0.001. Abbreviations: Entorhinal: entorhinal cortex; Fusiform: fusiform gyrus; MidTemp: middle temporal gyrus.

Significant group differences were observed in the ventricles, hippocampus, fusiform gyrus, middle temporal gyrus, and entorhinal cortex (Figure 2, Supplementary Table S1). Specifically, ventricle volume was largest in the pMCI group, whereas volumes of the other brain regions were smallest in this pMCI group. Notably, hippocampus effectively distinguished all three groups, while the remaining brain regions only differentiated the progressive (pMCI) group from the non-progressive (rMCI and sMCI).

### 3.4. Biomarker Characteristics of Participants

All biomarkers showed significant group differences (*p* < 0.001, Supplementary Table S2), indicating substantial variations in the underlying AD pathology levels among the three groups. Pairwise comparisons revealed that pMCI had the most pathological biomarker levels. Specifically, Aβ levels were lowest, while CSF tau and p-tau levels were highest in the pMCI group (*p*s < 0.001). In contrast, no significant differences were observed within the non-progressor cohort (rMCI vs. sMCI).

### 3.5. Partial Correlation Analysis

Partial correlation analyses were conducted for the entire sample, as well as separately within the rMCI, sMCI, and pMCI subgroups. Across the total sample, significant correlations were observed between cognitive performance and regional brain volumes after controlling for age, sex, education, *APOE ε4* status, and total intracranial volume, with all results surviving FDR correction. Specifically, larger ventricular volumes were associated with poorer cognitive performance, whereas greater volumes of the hippocampus, entorhinal cortex, fusiform gyrus, and middle temporal gyrus were associated with better cognitive function.

Subgroup analyses revealed that significant brain–behavior associations were predominantly observed in the sMCI and pMCI groups (*p*s < 0.05, FDR correction). In the sMCI group: ventricular volume was positively correlated with TMTB-A scores; hippocampal and entorhinal cortex volumes were negatively correlated with ADAS13 and positively correlated with RAVLT_delay and LDELtotal scores; fusiform gyrus volume showed significant correlations with LDELtotal, TMTB-A, and FAQ scores; middle temporal gyrus volume correlated positively with LDELtotal and CFT scores. In the pMCI group: hippocampal and entorhinal cortex volumes were significantly correlated with memory measures (RAVLT_delay, LDELtotal); the middle temporal gyrus volume was negatively correlated with TMTB-A scores. In contrast, in the rMCI group, no correlations reached statistical significance after correction (Supplementary Table S3).

### 3.6. Multinomial Logistic Regression Analysis of Predictive Factors

The optimized multinomial logistic regression model included ADAS-cog, RAVLT_immediate, FAQ, hippocampus, and fusiform volumes, using a data-driven forward entry approach. The model achieved a classification accuracy of 78%, with the sMCI group serving as the reference category.

**Figure 3:**
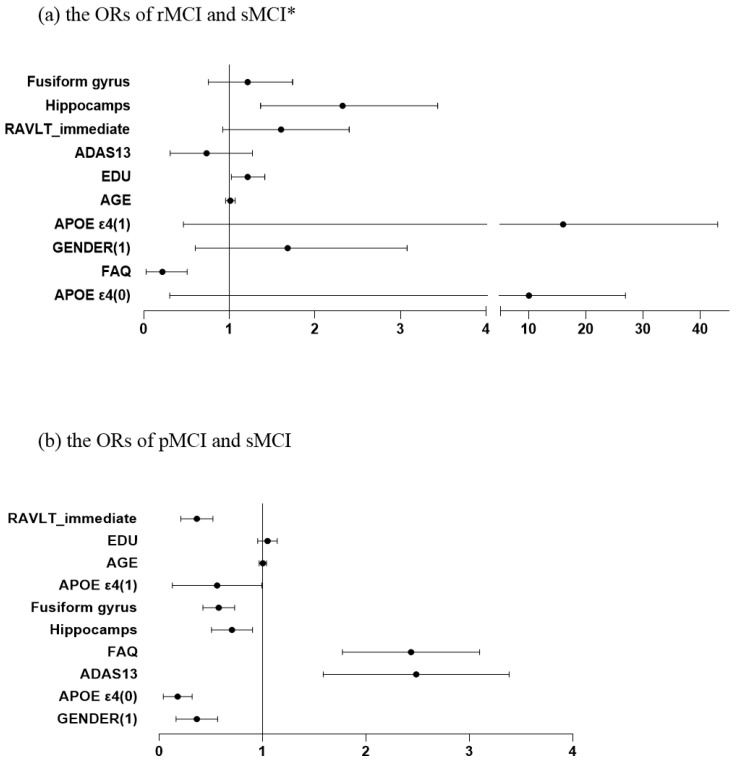
The odds ratios (ORs) for different variables. * X-axis segmented into intervals: [0, 4] [5, 45]. Abbreviations: Fusiform: fusiform gyrus; EDU: Years of education; ADAS: Alzheimer’s Disease Assessment Scale; FAQ: Functional Activities Questionnaire; RAVLT_immediate: Rey Auditory Verbal Learning Test (Immediate Recall).

Significant predictors of reversion from sMCI to rMCI were education, FAQ scores and hippocampal volume. Higher education, lower FAQ scores, and larger hippocampal volumes associated with an increased probability of reversion (Table 3, Figure 3a).

Significant predictors of progression from sMCI to pMCI were gender, RAVLT immediate recall scores, *APOE ε4 allele*, hippocampal volume, fusiform gyrus thickness, FAQ and ADAS-cog total scores. Among categorical predictors, male participants were less likely to progress to pMCI. *APOE ε4 alleles* of 0 or 1 was protective compared to 2 alleles. Among continuous predictors, higher ADAS-cog and FAQ scores increased the likelihood of progression, whereas Higher RAVLT-immediate recall scores reduced the risk of pMCI. Larger hippocampal and fusiform gyrus volumes were associated with a reduced risk of progression (Table 3, Figure 3b).

## 4. Discussion

This study demonstrates that subjects who converted within 3 years exhibited significant differences in baseline cognitive performance compared to non-converters. Among demographic variables, age distinguished the pMCI group, whereas educational attainment differentiated the rMCI group (Iraniparast et al., 2022)—suggesting that education plays a more critical role in the likelihood of MCI reversal.

Variables differentiating the three participant groups primarily involve general cognition, memory, daily functional ability, and language fluency. Specifically, performance on the ADAS-Cog and delayed recall measures effectively distinguished the three groups. Memory impairment represents a hallmark deficit in dementia. Research has consistently demonstrated that amnestic MCI (aMCI) has a higher progression rate to dementia than non-amnestic subtypes (Mitchell & Shiri-Feshki, 2009; Qin et al., 2023). However, despite the fact that this study’s population was predominantly aMCI (due to the enrollment criterion of objective memory impairment), we were able to observe significant differences in memory performance among participants. Our results lend additional support to the conclusion that individuals progressing to dementia within 36 months demonstrated markedly poorer baseline memory performance. Furthermore, participants who experienced cognitive reversal displayed superior baseline memory scores relative to other groups, further supporting the notion that memory plays a pivotal role in the transition of MCI. In addition, the FAQ differentiated the three groups, indicating that functional discrepancies are already present at baseline, despite MCI generally having minimal impact on daily functioning compared with dementia. Although these differences may not yet interfere with daily life, they are nonetheless detectable. Results from the CFT indicate that language functions are an influential factor in MCI progression. Although language impairment is not considered a core deficit in the early stages of AD, some studies have suggested that certain semantic tasks may serve as early cognitive markers during the prodromal phase of AD (Marra et al., 2021). Future research should further investigate the potentia role of language functions in MCI reversal.

Most other variables, including CDRSB, logical memory and TMT, distinguished pMCI from the general cohort but did not differentiate the two non-progressive groups. These findings suggest that pMCI and rMCI likely differ in their rates of cognitive decline, with pMCI typically exhibiting a more pronounced decrease in cognitive performance. Transitions from stability to progression are relatively common, whereas reversal is comparatively rare. Furthermore, although previous studies have identified risk factors including smoking history, alcohol consumption, obesity, hypertension, and a history of late-life depression—as influential factors in MCI progression (Durazzo et al., 2018; Marquié et al., 2023; Moody et al., 2021; W. Xu et al., 2017; Yu et al., 2024), our study did not observe significant differences. This discrepancy may be attributable to sample population, other unmeasured factors, the duration of follow-up, or the degree of variability (Johnson et al., 2021; Li et al., 2016), or associated with the degree of variability (Sundermann et al., 2017). Some risk factors may influence cognition differently depending on their intensity or duration, with effects emerging only beyond certain thresholds. Taking alcohol consumption as an example, studies have demonstrated that excessive drinking impairs cognitive function, whereas moderate intake is associated with better cognitive performance (W. Xu et al., 2017). Although obesity is recognized as a risk factor for dementia, elevated BMI has been suggested to confer metabolic reserve, potentially exerting a protective effect against MCI progression (Moody et al., 2021). Consequently, these factors may exhibit a non-linear relationship with MCI transition. Potential interactions among these factors may also exist. Previous studies have indicated that cognitive improvement requires the combined effects of multiple interventions, includes non-neurodegenerative factors (Ornish et al., 2024). Future investigations should adopt refined stratification approaches to disentangle the differential effects of distinct risk factor dimensions. Moreover, other potential factors, such as sleep disorders, were not considered in the analysis. Future research should therefore incorporate a broader set of factors to clarify their collective roles in MCI reversion.

Significant differences were observed across all brain regions; the pMCI group exhibited significantly larger ventricular volumes as well as reduced parenchymal volumes in the entorhinal cortex, medial temporal lobe, hippocampus, and fusiform gyrus compared to non-progressors, indicating distinct neurodegenerative patterns in pMCI. This finding is consistent with previous research demonstrating a close association between AD and brain atrophy (Li et al., 2016). However, neuroimaging data revealed that only hippocampal volume could differentiate all three groups, whereas other brain regions were effective solely in distinguishing progressive from non-progressive cases. Consistent with previous evidence demonstrating that preserved hippocampal volume predicts a higher likelihood of cognitive reversal (Dang et al., 2023), our findings confirmed this association. The hippocampus appears to be more vulnerable to cortical atrophy than other brain regions, and significant differences in cortical atrophy in non-hippocampal regions may only emerge during more advanced stages of the disease.

Our findings also suggest that caregivers are more perceptive of disease progression than patients themselves. Self-assessment tends to be biased, with both rMCI and pMCI individuals more likely to make conservative judgments about their condition, while caregivers are more sensitive to changes in assessment scores and better able to detect cognitive decline. These resluts align with prior research, which has consistently demonstrated an association between caregiver sensitivity and accurate detection of disease progression (Manjavong et al., 2024; Thomas et al., 2024). Studies have shown significant discrepancies between caregiver and patient assessments, particularly in evaluating social functioning, with caregivers often providing more accurate distinctions in severity levels (Rangel et al., 2025). Furthermore, the severity of patient symptoms is positively correlated with the burden experienced by caregivers (Rebolo et al., 2025). Future research and clinical practice should place greater emphasis on caregiver-reported assessments of patients’ functional and cognitive status. Allocating increased attention and resources to caregivers is crucial for enhancing patient quality of life, improving the predictability of disease progression, and safeguarding the psychological well-being of the caregivers themselves.

Nearly all participants exhibited significant associations between brain regions and cognitive measures in the partial correlation analyses, This finding is consistent with previous research highlighting the close relationship between brain structure and cognitive function (Zhang et al., 2021). Interestingly, in the subgroup analyses, a greater number of significant correlations were observed in the sMCI group. This may be partly explained by the larger sample size in this group. Alternatively, the relationship between brain volume changes and cognition may not follow a strictly linear trajectory, and potential inflection points could exist. Another possible explanation is that sMCI individuals possess more stable neuroanatomical features compared to the other two groups, whose brain regions may be undergoing more latent dynamic changes. This finding highlights a potentially overlooked aspect: sMCI may, in certain respects, more clearly reflect the underlying pathological characteristics of MCI. Future studies would benefit from employing segmented or stratified analytical approaches to further elucidate this phenomenon.

In the final model, factors significantly associated with cognitive improvement were educational attainment, daily functional ability, and hippocampal volume. In contrast, progression was influenced by gender, immediate recall ability, the number of *APOE4 alleles*, ADAS-cog score, and the volumes of the hippocampus and fusiform gyrus. These well-established risk factors for progression were confirmed in our study. The differences in predictive variables between the two models underscore the distinct mechanisms underlying progression and reversal.

Although gender did not differ significantly across groups, the model indicated that males are less likely to progress to pMCI, a finding partially supported by prior research (Altmann et al., 2014). It is noteworthy that previous studies have found the progression rates differ between males and females across various age ranges (Bun et al., 2023; Mian et al., 2024). This may be attributable to inherent biological factors, such as hormonal fluctuations, socio-cognitive differences between genders or interaction effects with other factors. The remaining variables were consistent with previous findings. Notably, age—typically regarded as a major risk factor for MCI progression (M. Wang et al., 2023)—did not emerge as a significant predictor in the model. This unexpected result may be explained by severa factors. First, age may exhibit collinearity with other variables (such as disease-related or protective factors). Second, the relatively short follow-up period and narrow age range of the cohort may have reduced the statistical power to detect age-related effects (Malek-Ahmadi, 2016).

This study also has several limitations that should be acknowledged. (1) The rMCI group had a small sample size, which may lead to some potential differences that are difficult to detect. Nevertheless, these cases were identified from a large-scale database, and their prevalence aligns with previously reported epidemiological estimates. Furthermore, we implemented stringent screening criteria for participants, ensuring that the reversed cognitive status remained stable throughout the follow-up period. Future studies should aim to increase the sample size of rMCI participants to improve statistical power. (2) The neuroimaging analysis was was restricted to volumetric comparisons of known brain region, without a comprehensive investigation of neuroimaging biomarkers. Future studies should place greater emphasis on exploring neuroimaging biomarkers, particularly structural changes in the hippocampal and peri-hippocampal. (3) Due to sample size limitations, while biomarkers can reveal the underlying pathological status of participants, no significant associations were found between the biomarkers and cognitive reversal status in this study. While our between-group analyses elucidated their role in disease progression, future studies are warranted to further investigate the reversion of it. This study utilized CSF biomarkers, future research could explore other types of biological markers. (4) Given the need to establish temporally precise predictors for targeted early intervention, this study adopted a 36-month follow-up period to optimally capture critical transition windows in disease progression. However, the follow-up remains relatively short, and longitudinal changes beyond this period were not assessed. Future studies should extend the follow-up duration to evaluate the long-term effects of various contributing factors. (5) Our study revealed that even among MCI participants with baseline memory impairment, memory continues to exhibit predictive value. However, due to the limitations of the ADNI dataset, we were unable to examine the role of other cognitive impairments in reversion. Future studies should prioritize targeted analyses based on aMCI and naMCI subtypes to further refine and validate these findings. (6) Cultural background is a meaningful variable that cannot be overlooked in studies of MCI progression, as it may influence symptom perception, diagnostic processes, or healthcare utilization. Due to limitations of the ADNI dataset—specifically the lack of direct cultural assessment indicators (e.g., cultural values, language-related factors)—we were unable to conduct in-depth analyses of cultural influences in our study. Future research should consider cross-cultural cohort studies that integrate culturally specific assessment tools to clarify the extent to which cultural factors modulate MCI progression.

## Figures and Tables

**Table 1:** Demographic characteristics and behavior data.

			rMCI	sMCI	pMCI	F/χ^2^	*p*-Value
**Demographic Characteristics**		N	52	432	272		
	Age, years	Range	55–89.3	55–90	55–88.3	11.667	0.003
		Mean	70.94 ± 8.22	72.37 ± 7.57	73.84 ± 7.03	5.012	0.007 de
	Sex, male/female		28/24	265/167	156/116	1.814	0.404
		N	52	432	272		
	Education, years	Range	12–20	7–20	6–20	9.038	0.011
		Mean	17.08 ± 2.27	16.06 ± 2.73	15.82 ± 2.77	4.663	0.01 ac
	*APOE ε4*, 0/1/2		29/22/1	261/139/32	87/140/45	60.458	<0.001
**General Cognition**	CDRSB	N	52	432	272	59.542	<0.001 de
		Mean	1.10 ± 0.79	1.28 ± 0.76	1.98 ± 0.97		
	ADAS13	N	52	429	270	136.749	<0.001 def
		Mean	10.60 ± 3.93	14.13 ± 5.63	21.45 ± 5.95		
	MMSE	N	52	432	272	39.681	<0.001 abc
		Mean	28.73 ± 1.37	27.97 ± 1.67	26.78 ± 1.82		
**Memory**	RAVLT_immediate	N	52	432	272	103.901	<0.001 abc
		Mean	43.29 ± 9.37	37.29 ± 10.12	27.83 ± 7.13		
	RAVLT_delay	N	52	432	272	77.579	<0.001 abc
		Mean	6.90 ± 3.73	4.90 ± 3.87	1.69 ± 2.32		
	LDELtotal	N	52	432	272	96.446	<0.001 ab
		Mean	8.13 ± 2.53	6.91 ± 3.18	3.51 ± 3.05		
**Executive Function**	TMTB-A	N	52	432	272	38.964	<0.001 de
		Mean	43.37 ± 21.75	61.00 ± 40.64	98.57 ± 71.58		
**Daily Function**	FAQ	N	52	426	270	94.7	<0.001 def
		Mean	0.56 ± 0.98	1.92 ± 3.06	5.76 ± 4.93		
**Language Fluency**	CFT	N	52	432	272	34.907	<0.001 abc
		Mean	20.56 ± 4.70	18.27 ± 5.00	15.21 ± 4.70		
**Lifestyle**	Married or living with a partner, No/Yes/Unknown		12/40/0	98/329/5	51/221/0	5.61	0.23
	BMI	N	52	432	272		
		Mean	27.91 ± 5.79	27.57 ± 4.59	26.32 ± 4.62	6.734	0.001 b
	Obesity, No/Yes		31/21	270/162	196/76	7.702	0.021
	Smoking, No/Yes		23/18	236/137	148/109	2.44	0.295
	Drug, No/Yes		40/1	370/3	257/0	4.164	0.125
	Alcohol, No/Yes		40/1	364/9	244/13	3.346	0.188
**Clinical Characteristics**	Psychiatric, No/Yes		30/22	269/163	168/104	0.412	0.814
	Neurologic, No/Yes		34/18	272/160	199/73	7.878	0.019
	Cardiovascular, No/Yes		17/35	136/296	84/188	0.075	0.963
	Hypertension, No/Yes		28/24	230/202	133/139	1.362	0.506
	Stroke, No/Yes		52/0	429/3	264/8	6.7	0.035
	Respiratory, No/Yes		41/11	219/53	319/113	4.303	0.116
	Endocrine-Metabolic, No/Yes		28/24	253/179	157/115	0.432	0.806
	Malignancy, No/Yes		44/8	336/96	207/65	1.833	0.4
	Major Surgical Procedures, No/Yes		15/26	84/289	71/186	5.019	0.081
	GDS	N	52	432	271		
		Mean	1.19 ± 1.34	1.66 ± 1.44	1.69 ± 1.42	2.753	0.064

^a^: rMCI > pMCI; ^b^: sMCI > pMCI; ^c^: rMCI > sMCI; ^d^: rMCI < pMCI; ^e^: sMCI < pMCI; ^f^: rMCI < sMCI; Abbreviations: rMCI: Reversible Mild Cognitive Impairment; sMCI: Stable Mild Cognitive Impairment; pMCI: Progressive Mild Cognitive Impairment; CDRSB: Clinical Dementia Rating Scale Sum of Boxes; ADAS: Alzheimer’s Disease Assessment Scale; MMSE: Mini-Mental State Examination; RAVLT_immediate: Rey Auditory Verbal Learning Test (Immediate Recall); RAVLT_delay: Rey Auditory Verbal Learning Test Delayed Recall; LDELtotal: Logical Memory Delayed Recall Total; TMTB-A: Trail Making Test Part B Score-Trail Making Test Part A Score; FAQ: Functional Activities Questionnaire; CFT: Category Fluency (Animal Naming) Score.

**Table 2:** Everyday cognitive performance of three groups.

	EcogPt			EcogSP			Group	Ecog	Group × Ecog
	rMCI	sMCI	pMCI	rMCI	sMCI	pMCI	F(*p*)	F(*p*)	F(*p*)
**Memory**	2.08 ± 0.68	2.29 ± 0.69	2.48 ± 0.72	1.63 ± 0.51	2.03 ± 0.71	2.84 ± 0.81	36.314	0.267	21.295
							(<0.001)	(0.606)	(<0.001)
**Language**	1.76 ± 0.64	1.88 ± 0.64	1.94 ± 0.70	1.26 ± 0.40	1.55 ± 0.59	2.11 ± 0.83	18.913	0.333	16.141
							(<0.001)	(0.564)	(<0.001)
**Visual-Spatial**	1.36 ± 0.52	1.42 ± 0.55	1.58 ± 0.65	1.18 ± 0.23	1.32 ± 0.47	1.80 ± 0.77	20.817	1.101	7.264
							(<0.001)	(0.295)	(0.001)
**Plan**	1.31 ± 0.48	1.48 ± 0.58	1.66 ± 0.65	1.21 ± 0.41	1.47 ± 0.62	1.93 ± 0.82	20.98	0.64	4.766
							(<0.001)	(0.424)	(0.009)
**Organization**	1.51 ± 0.63	1.58 ± 0.65	1.72 ± 0.75	1.31 ± 0.55	1.51 ± 0.67	2.14 ± 0.89	20.791	1.932	11.66
							(<0.001)	(0.165)	(<0.001)
**DividedAttention**	1.95 ± 0.77	1.98 ± 0.82	1.99 ± 0.78	1.57 ± 0.58	1.79 ± 0.78	2.38 ± 0.94	10.112	0.377	11.507
							(<0.001)	(0.54)	(<0.001)
**Total**	1.69 ± 0.51	1.79 ± 0.54	1.92 ± 0.59	1.36 ± 0.36	1.62 ± 0.53	2.22 ± 0.70	31.297	0.497	17.96
							(<0.001)	(0.481)	(<0.001)

Abbreviations: EcogPt: Everyday Cognition—Participant Version; EcogSP: Everyday Cognition—Study Partner Version.

**Table 3:** Multivariate logistic regression analysis of groups.

Variable	rMCI/sMCI			pMCI/sMCI		
	β	OR (95%CL)	*p*	β	OR (95%CL)	*p*
AGE	0.011	1.011	0.707	0.0005	1.0005	0.98
GENDER, Male	0.308	1.361	0.459	−1.125	0.325	<0.001
EDU	0.185	1.204	0.024	0.042	1.043	0.363
*APOE ε4*, 0	1.051	2.86	0.358	−1.961	0.141	<0.001
*APOE ε4*, 1	1.498	4.472	0.195	−0.805	0.447	0.061
ADAS13	−0.468	0.626	0.195	0.866	2.377	<0.001
FAQ	−2.133	0.118	0.004	0.865	2.374	<0.001
RAVLT_immediate	0.398	1.489	0.102	−0.175	0.341	<0.001
Hippocampus	0.773	2.166	0.001	−0.38	0.684	0.009
Fusiform gyrus	0.136	1.146	0.521	−0.579	0.561	<0.001

Abbreviations: EDU: Years of education; ADAS: Alzheimer’s Disease Assessment Scale; FAQ: Functional Activities Questionnaire; RAVLT_immediate: Rey Auditory Verbal Learning Test (Immediate Recall).

## Data Availability

All data are available upon reasonable request or can be obtained from the Alzheimer’s Disease Neuroimaging Initiative (ADNI) database (adni.loni.usc.edu).

## Supplementary Materials

The following supporting information can be downloaded at: https://www.mdpi.com/article/10.3390/bs15111552/s1, Table S1: Group differences in brain region volumes among the three MCI subtypes; Table S2: Group differences in biomarker characteristics among the three MCI subtypes; Table S3: Correlation between brain regions and cognitive measure.

## Abbreviations

The following abbreviations are used in this manuscript:

MCI	mild cognitive impairment
pMCI	progressive mild cognitive impairment
rMCI	reversible mild cognitive impairment
sMCI	stable mild cognitive impairment
APOE	apolipoprotein E
CDRSB	Clinical Dementia Rating Scale Sum of Boxes
ADAS	Alzheimer’s Disease Assessment Scale
MMSE	Mini-Mental State Examination
RAVLT_immediate	Rey Auditory Verbal Learning Test (Immediate Recall)
RAVLT_delay	Rey Auditory Verbal Learning Test Delayed Recall
LDELtotal	Logical Memory Delayed Recall Total
TMTB-A	Trail Making Test Part B Score- Trail Making Test Part A Score
FAQ	Functional Activities Questionnaire
CFT	Category Fluency (Animal Naming) Score.
EcogPt	Everyday Cognition Scale—Participant Version
EcogSP	Everyday Cognition Scale—Study Partner Version
EDU	Years of education
Entorhinal	entorhinal cortex
Fusiform	fusiform gyrus
MidTemp	middle temporal gyrus
