# Supplementary material for: Predictors of Transition from Mild Cognitive Impairment to Normal Cognition and Dementia
Source: Behav Sci (Basel). 2025 Nov 14;15(11):1552. doi: 10.3390/bs15111552 (PMC12649382; doi:10.3390/bs15111552)
Supplement: Supplementary file 1 [file behavsci-15-01552-s001.zip › behavsci-3821468-supplementary.pdf]

Supplementary Table 1 Group differences in brain region volumes among the three MCI subtypes.

		rMCI	sMCI	pMCI	F	P-value
Ventricles	N	50	414	265	15.379	<0.001[de]
	Mean	31473.27±17855.41	38110.69±21510.84	44770.74±21686.46		
Hippocampus	N	44	374	228	52.287	<0.001[abc]
	Mean	7708.59±864.21	7149.21±1060.52	6174.76±1025.64		
Entorhinal cortex	N	43	371	230	30.18	<0.001[ab]
	Mean	3989.56±594.00	3776.33±747.15	3154.91±741.85		
Fusiform gyrus	N	43	371	230	21.162	<0.001[ab]
	Mean	18493.81±2040.05	18441.83±2681.79	16359.13±2655.25		
Middle temporal gyrus	N	43	371	230	34.455	<0.001[ab]
	Mean	20858.79±2637.81	20655.53±2690.14	18188.63±2903.92		

a:rMCI>pMCI b:sMCI>pMCI c:rMCI>sMCI

d:rMCI<pMCI e:sMCI<pMCI f:rMCI<sMCI

Supplementary Table 2 Group differences in biomarker characteristics among the three MCI subtypes.

		rMCI	sMCI	pMCI	F	P-value
Aβ	N	18	234	169	18.334	<0.001[ab]
	Mean	1003.94±340.42	930.51±356.62	681.36±258.89		
tau	N	30	289	172	24.939	<0.001[de]
	Mean	215.09±65.64	260.20±109.88	354.63±136.55		
P-tau	N	30	289	172	26.494	<0.001[de]
	Mean	19.33±7.11	24.71±12.24	35.62±15.65		

a:rMCI>pMCI   b:sMCI>pMCI   c:rMCI>sMCI

d:rMCI<pMCI   e:sMCI<pMCI   f:rMCI<sMCI

Supplementary Table 3 Correlation between brain regions and cognitive measure

		Ventricles	Hippocampus	Entorhinal cortex	Fusiform gyrus	Middle temporal gyrus
CDRSB	rMCI	0.143	0.171	-0.266	-0.182	0.014
	sMCI	0.121	-0.078	-0.082	-0.134	-0.1
	pMCI	0.091	-0.033	-0.046	0.042	0.047
	total	0.171***	-0.166***	-0.157***	-0.133**	-0.128**
ADAS13	rMCI	-0.097	-0.051	-0.082	-0.21	-0.229
	sMCI	0.095	-0.272***	-0.273***	0.029	-0.115
	pMCI	0.071	-0.205*	-0.131	-0.05	-0.146
	total	0.168***	-0.386***	-0.323***	-0.139***	-0.269***
MMSE	rMCI	-0.059	-0.094	0.401	0.24	0.058
	sMCI	-0.132	0.147	0.109	0.079	0.051
	pMCI	0.029	0.037	-0.024	0.038	0.101
	total	-0.119***	0.202***	0.161***	0.145***	0.169***
RAVLT_immediate	rMCI	0.171	0.039	-0.035	-0.045	0.197
	sMCI	0.008	0.094	0.147	-0.025	0.004
	pMCI	-0.019	0.066	-0.024	-0.058	0.103
	total	-0.087*	0.24***	0.197***	0.083*	0.18***
RAVLT_delay	rMCI	0.166	0.198	0.015	0.005	0.165
	sMCI	-0.114	0.265***	0.216***	-0.005	0.049
	pMCI	-0.027	0.299***	0.187*	-0.149	0.025
	total	-0.139***	0.369***	0.277***	0.057	0.155***
LDELtotal	rMCI	0.097	-0.026	0.107	0.069	0.211
	sMCI	-0.03	0.213***	0.227***	0.175**	0.148*
	pMCI	-0.052	0.288***	0.227**	-0.033	0.091
	total	-0.113**	0.355***	0.314***	0.187***	0.238***
TMTB-A	rMCI	0.188	0.092	-0.263	0.176	0.081
	sMCI	0.206***	-0.021	-0.038	-0.145*	-0.047
	pMCI	0.075	0.072	0.071	-0.108	-0.215**
	total	0.179***	-0.09*	-0.076	-0.172***	-0.199***
FAQ	rMCI	0.121	0.021	-0.033	-0.038	0.184
	sMCI	0.108	-0.1	-0.011	-0.147*	-0.188
	pMCI	0.059	0.047	0.038	0.063	0.031
	total	0.159***	-0.185***	-0.113**	-0.138***	-0.186***
CFT	rMCI	-0.049	-0.033	-0.449	0.019	-0.03
	sMCI	-0.102	0.149*	0.055	0.047	0.135
	pMCI	-0.032	-0.092	-0.015	0.087	0.154
	total	-0.123***	0.159***	0.09*	0.123**	0.204***

Abbreviations:

ADAS: Alzheimer's Disease Assessment Scale; RAVLT_immediate: Rey Auditory Verbal Learning Test (Immediate Recall);RAVLT_delay: Rey Auditory Verbal Learning Test Delayed Recall; LDELtotal: Logical Memory Delayed Recall Total; TMTB-A: Trail Making Test Part B Score- Trail Making Test Part A Score; FAQ: Functional Activities Questionnaire; CFT: Category Fluency (Animal Naming) Score

$^{*}p<0.05$, $^{**}p<0.01$, $^{***}p<0.001$
